# Fabrication of High-Resolution Fine Microneedles Derived from Hydrolyzed Hyaluronic Acid Gels in Vacuum Environment Imprinting Using Water Permeable Mold

**DOI:** 10.3390/gels8120785

**Published:** 2022-11-29

**Authors:** Sayaka Miura, Rio Yamagishi, Riku Miyazaki, Kaori Yasuda, Yuki Kawano, Yoshiyuki Yokoyama, Naoto Sugino, Takao Kameda, Satoshi Takei

**Affiliations:** 1 Department of Pharmaceutical Engineering, Toyama Prefectural University, Imizu, Toyama 939-0398, Japan; 2 Toyama Industrial Technology Research and Development Center, Takaoka, Toyama 933-0981, Japan; 3 Futuristic Technology Department, Sanko Gosei, Nanto, Toyama 939-1852, Japan

**Keywords:** fine microneedles, hydrolyzed hyaluronic acid, functional gels, cosmetics, transdermal drug delivery system, water permeable mold, imprint lithography

## Abstract

Hydrolyzed hyaluronic acid high-resolution fine microneedles of 13 µm in diameter and 24 µm in height were fabricated from hydrolyzed hyaluronic acid gels made in mixtures of water using vacuum environment imprint lithography processes with a water permeable mold. The gas traps of water and volatile solvents in the imprint materials cause transfer failure in the conventional water impermeable molds of quartz and metal. However, the water permeable mold allows the use of 67 wt% dilution water with high solubility to increase the fluidity of the hydrolyzed hyaluronic acid during the patterning of high-resolution fine microneedles for cosmetics and pharmaceuticals. This demonstration sets a new paradigm of functional pure gels for high-resolution nano-patterning applications with various cosmetic and pharmaceutical materials containing dilution water using a water permeable mold.

## 1. Introduction

Many beauty ingredients, such as hyaluronic acid and collagen gels, are expected to bring significant demand to the global market for cosmetology products [1]. Hyaluronic acid made in mixtures of solvents has excellent water retention properties and can contain approximately 1000 times its weight in water [2,3]. However, the concentration of hyaluronic acid, which occurs naturally in the skin, declines with age, affecting wrinkle formation, skin elasticity, and dryness [4,5]. Therefore, hyaluronic acid can be used in cosmetic products to provide anti-ageing and anti-wrinkle benefits [6,7].

The skin is composed of an epidermal layer, a dermal layer, and subcutaneous tissue, with a stratum corneum on the surface of the epidermal layer. Since the stratum corneum has a barrier function to prevent foreign substances from entering the body and moisture evaporation from the body [8,9], high-molecular-weight cosmetic ingredients such as hyaluronic acid cannot be delivered into the skin via transdermal administration [10,11]. Various methods have been used to determine how to penetrate the stratum corneum to the epidermis, i.e., the deep layer within the skin [12]. For example, hypodermic needles have been used, but they are painful and many patients have needle phobias; moreover, these needles require knowledgeable medical personnel to administer them [13,14].

Currently, technologies such as nano-emulsions, liposomes, nanoparticles, and other nanotechnologies have been developed to penetrate the stratum corneum with active ingredients in cosmetics [15,16]. These methods have shown significant improvement in the skin retention of active ingredients and are expected to enhance skin health. Microneedles are one of these technologies that are expected to be able to deliver water-absorbing ingredients safely and efficiently [17,18,19,20,21,22]. Microneedles shaped by microfabrication come in a variety of types, but soluble microneedles including hyaluronic acid and collagen gels have been used in a wide range of cosmetic and transdermal drug delivery system applications. The microneedles have the benefit of enhancing penetration, eliminating the risk of needles remaining in the body or leaving sharp waste that could be a biohazard after use [23,24].

Conventional microneedles for cosmetic applications are 100–800 µm in diameter and 200–800 µm in height. Conversely, the epidermal layer is approximately 200 µm thick. Consequently, the microneedles reach the dermis layer, which exists below the epidermis layer, leading to a painful injection because the dermal layer contains sensory receptors [25].

However, the microfabrication process involves problems such as gas generation from water and solvents during heating, molding defects, and mold breakage [26,27,28]. Furthermore, the microfabrication of a hyaluronic acid gel solution containing high concentrations of water and volatile solvents was difficult because of its high viscosity in solution even at low concentrations [29,30,31,32]. To improve the resolution of microneedles and achieve miniaturization, we developed gas and water permeable molds that can vent the gas generated during heat molding [33,34,35]. The high-resolution fine microneedle pattern failure of hydrolyzed hyaluronic acid gels containing 67 wt% water as a dilution solvent was demonstrated using the water permeable mold and the vacuum environment imprint lithography processes for skin care cosmetic patches.

## 2. Results and Discussion

**Figure 1:**
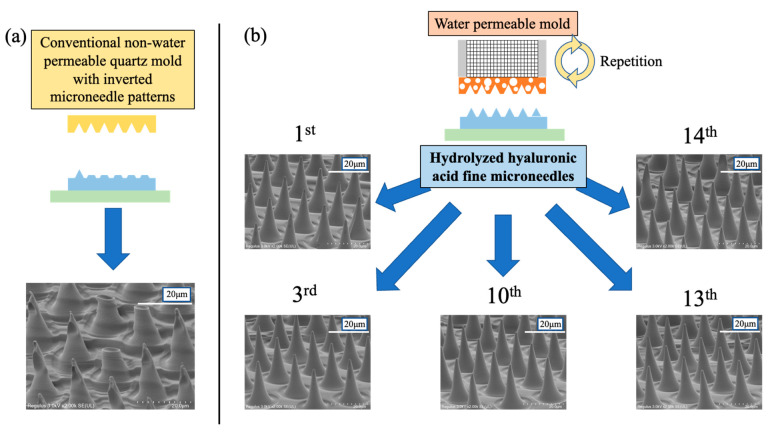
SEM images of hydrolyzed hyaluronic acid fine microneedles fabricated at (**a**) 1st imprint by using conventional non-water permeable quartz mold with inverted microneedle patterns and (**b**) 1st, 3rd, 10th, 13th, and 14th imprints by using water permeable mold in vacuum environment imprint lithography processes.

Figure 1 shows the scanning electron microscope (SEM) images of the hydrolyzed hyaluronic acid fine microneedles fabricated at (a) the 1st imprint by using a conventional non-water permeable quartz mold with inverted microneedle patterns and (b) the 1st, 3rd, 10th, 13th, and 14th imprints by using the water permeable mold in the vacuum environment imprint lithography processes. There is no significant difference in patterning defects between the 1st and 14th imprints using the same water permeable mold. The hydrolyzed hyaluronic acid solutions were filled into the tip of the needles in the water permeable mold, and patterning defects were improved by using (b) the water permeable mold compared with (a) the conventional non-water permeable quartz mold. This is because the water vapor from the water evaporation generated during the processes permeated through the water permeable mold, improving the filling rate of the highly viscous hydrolyzed hyaluronic acid into the water permeable mold.

The high-resolution fine microneedles of approximately 13 µm in diameter and 24 µm in height, with hydrolyzed hyaluronic acid and water as their two components, were fabricated using the newly vacuum environment imprint lithography processes using a water permeable mold for cosmetic and pharmaceutical applications. The prepared water permeable surface material in the water permeable mold with titanium dioxide and silicon dioxide were cross-linked by the conditions to generate the cross-link density needed to obtain water permeability. The possibility of shortening the molding time by further improving the filling speed is expected to be realized in the next study.

**Figure 2:**
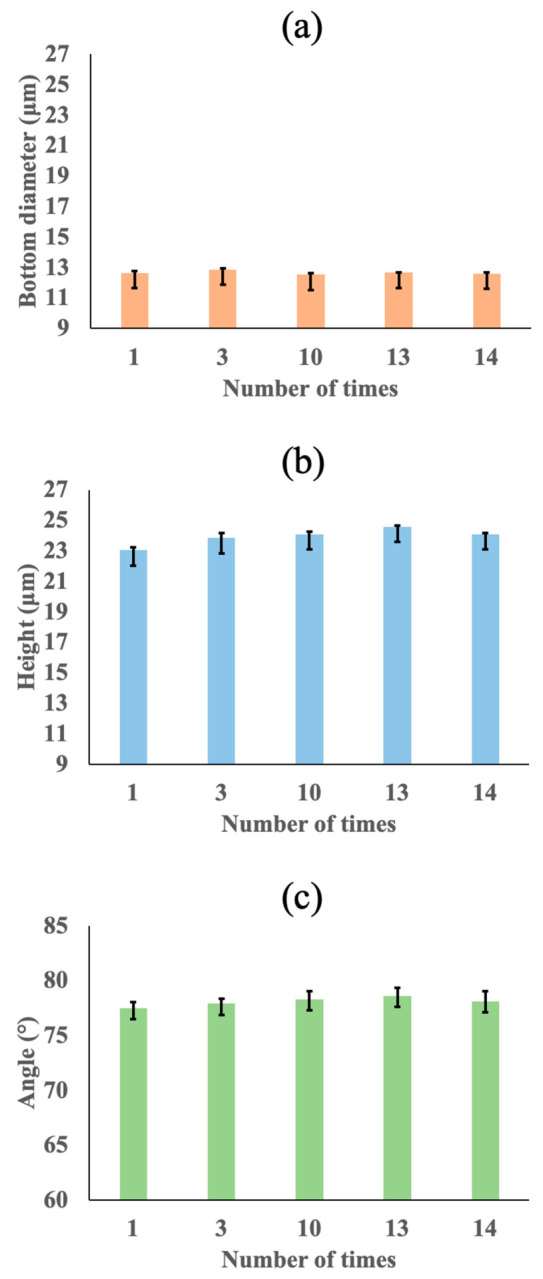
Measurement results of (**a**) bottom diameter, (**b**) height, and (**c**) angle of microneedle patterns obtained by calculating sharpness of SEM images on fabricated hydrolyzed hyaluronic acid fine microneedles at 1st, 3rd, 10th, 13th, and 14th imprints.

In addition, Figure 2 shows the measurement results of (a) the bottom diameter, (b) the height and (c) the angle of the microneedle patterns by calculating the sharpness of the SEM images on the fabricated hydrolyzed hyaluronic acid fine microneedles at the 1st, 3rd, 10th, 13th, and 14th imprints. Although the measured result of the bottom diameter (Standard deviation; 0.11 µm), height (Standard deviation; 0.48 µm) and angle (Standard deviation; 0.37°) of microneedle patterns was considered to be a data variability by calculating the sharpness of the SEM images in five different areas, the size biases of the microneedles do not affect the above application of cosmetic and pharmaceutical microneedles. These results indicated that the water permeable mold improved the patterning defects of the hydrolyzed hyaluronic acid gel solutions containing high concentrations of 67 wt% water and can be expected to be useful for the mass production of high-resolution fine microneedles using production-level automatic imprint lithography equipment.

**Figure 3:**
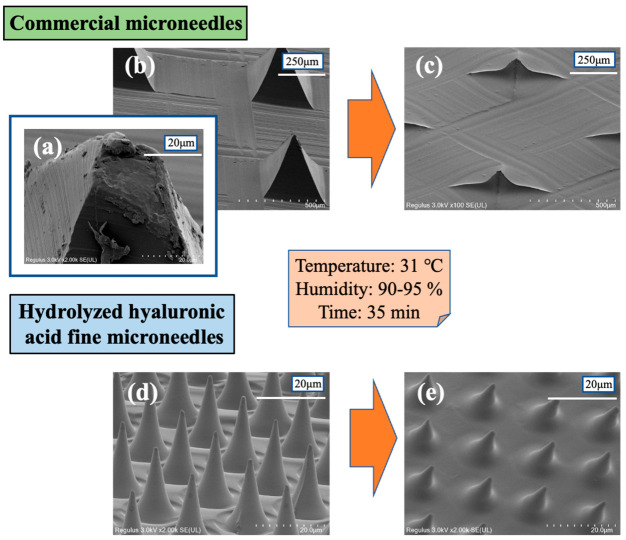
Comparison of microneedles size and dissolution behavior of commercial microneedles and imprinted hydrolyzed hyaluronic acid fine microneedles.

Figure 3 is a comparison of the microneedle sizes and the dissolution behavior of (a,b) commercial microneedles (TAPPULEENA, JINBEEKOA, Japan) of 364 µm in the length on one side of the bottom of the rhombus and 346 µm in height and (d) the imprinted hydrolyzed hyaluronic acid fine microneedles of approximately 13 µm in diameter and 24 µm in height. The tip of the above commercial microneedle was not pointed and appeared to be flat compared with the tip of the newly developed hydrolyzed hyaluronic acid fine microneedles. Both (e) the hydrolyzed hyaluronic acid fine microneedles without additives and (c) the commercial microneedles with sodium hyaluronate, as well as various additives such as glycerine and butylene glycol, which have the hygroscopic properties to dissolve easily under high humidity conditions, had more than 60% of their volume dissolve after 35 min. The hydrolyzed hyaluronic acid fine microneedles were advantageous in that they acted in the skin within a shorter time, broadening the application areas of the high-resolution microneedles to cosmetics and pharmaceuticals.

**Figure 4:**
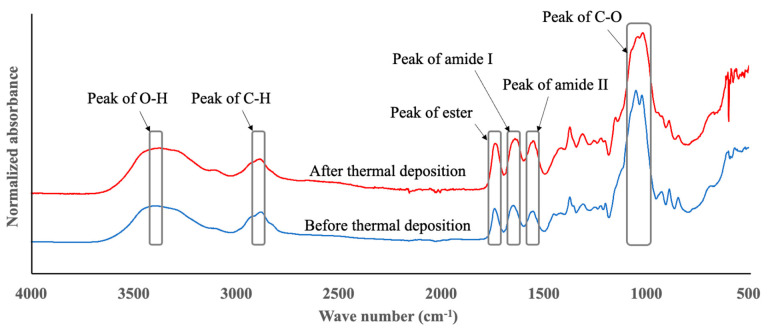
FT-IR spectra of hydrolyzed hyaluronic acid before and after thermal deposition for evaluating thermal decomposition in fine microneedle fabrication.

Figure 4 shows the Fourier transform infrared spectrometer (FT-IR) spectra of the hydrolyzed hyaluronic acid before and after thermal deposition for evaluating the thermal decomposition in fine microneedle fabrication. The 3400 cm^−1^ (OH bond), 2880 cm^−1^ (CH bond), 1740 cm^−1^ (ester), 1649 cm^−1^ (amide type I), 1554 cm^−1^ (amide type II), and 1045 cm^−1^ (CO bond) peaks were observed before thermal deposition, and 3375 cm^−1^ (OH bond), 2885 cm^−1^ (CH bond), 1735 cm^−1^ (ester), 1640 cm^−1^ (amide type I), 1551 cm^−1^ (amide type II), and 1033 cm^−1^ (CO bond) peaks after thermal deposition also indicated the absorption of hydrolyzed hyaluronic acid and water, and these peaks were somewhat coincident before and after thermal deposition at 80 °C for 90 min.

**Figure 5:**
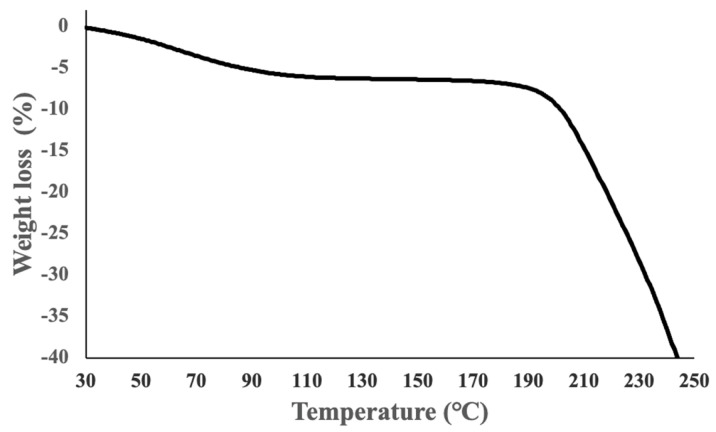
Tg-DTA curve of hydrolyzed hyaluronic acid in nitrogen.

Figure 5 shows the thermos-gravimetric differential thermal analysis instrument (Tg-DTA) curve of the hydrolyzed hyaluronic acid. During heating up to approximately 100 °C, a continuous weight loss occurs nearly imperceptibly owing to the removal of the water in the hydrolyzed hyaluronic acid. No shoulder and no sharp signal can be observed up to approximately 170 °C in the hydrolyzed hyaluronic acid. The hydrolyzed hyaluronic acid in the microneedles was suggested to be stable up to 170 °C. Therefore, the above adjusted conditions at 40 °C for 15 min in the vacuum environment imprint lithography processes were also acceptable for the fabrication of hydrolyzed hyaluronic acid fine microneedles with high-resolution.

A more accurate evaluation of the biocompatibility such as vivo penetration experiments on mice and skin penetration is the subject of a future study. The result that one can nanoimprint functional gel solutions containing high volatile dilution solvents of over 60% is expected to be a breakthrough for various biomedical applications.

## 3. Conclusions

The high-resolution fine microneedle pattern failure of hydrolyzed hyaluronic acid gels containing 67 wt% water as a dilution solvent was solved using the water permeable mold in the vacuum environment imprint lithography processes. This demonstration of the high-resolution fine microneedles of 13 µm in diameter and 24 µm in height sets a new paradigm of functional pure gels for high-resolution fine patterning applications with various cosmetic and pharmaceutical materials containing dilution water using a water permeable mold for a skin care cosmetic patch and transdermal drug delivery system.

## 4. Materials and Methods

**Figure 6:**
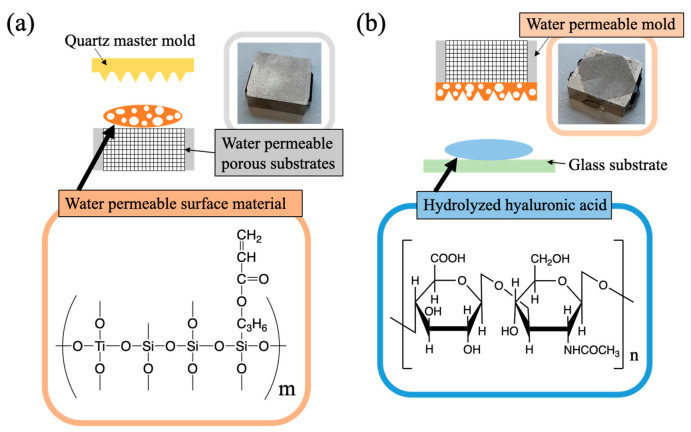
Chemical structures of (**a**) water permeable surface material and (**b**) hydrolyzed hyaluronic acid as a raw material of fine microneedles.

Figure 6 shows (a) the chemical structures of the water permeable surface material and (b) hydrolyzed hyaluronic acid as a raw material of fine microneedles. The water permeable surface materials with four components, 40 wt% 3-(acryloyloxy)propyltrimethoxysilane, 35 wt% methyltrimethoxysilane, 15 wt% tetraethyl titanate, and 10 wt% tetraethoxysilane, were prepared using the sol–gel polymerization [36,37,38]. The polymerized water permeable surface materials with an average molecular weight of 5500 relative to a polystyrene standard were measured using gel permeation chromatography (GPC, EcoSEC Elite HLC-8420GPC, Tosoh, Japan). GPC was performed using connected separation columns: SuperAW-H columns (TSK-GEL, Tosoh, Japan); N, N-dimethylformamide was used as the mobile phase. The solvent flow rate was fixed to 0.6 mL/min and the column temperature was maintained at a constant 40 °C. The 90 wt% water permeable surface material and 10 wt% cross-linkers were blended and stirred for 3 min. Then, the water permeable surface material was fabricated, after defoaming for 2 h in a vacuum dryer (AVO-250SB, AS ONE, Japan).

The water permeable porous substrates under the water permeable surface material were fabricated by using a hybrid metal 3D printer (LUMEX Avance-25, Matsuura, Japan) and the standard maraging steel powders with an average particle size of 20–30 μm. The powders were baked and hardened by irradiating them with a 400 W Yb fiber laser using the 3D printer [39,40]. This series of operations was repeated 10 times, and the shape was cut using a cutting process. The above water permeable surface material was dispensed on the surface of the water permeable porous substrates and polymerized at 180 °C for 20 min.

The hydrolyzed hyaluronic acid (Hyalo-Oligo, Kewpie, Japan) shown in Figure 6b was used as the only solid content of the fine microneedles with hydrolyzed hyaluronic acid and water as their two components. The concentration of the hydrolyzed hyaluronic acid gel solutions had a trade-off relationship between the fluidity of the high-resolution nanoimprint transfer material and the process time of the vacuum environment imprinting using a water permeable mold. The 33 wt% hydrolyzed hyaluronic acid as the adjusted hydrolyzed hyaluronic acid gel solutions was mixed with 67 wt% pure water at 35 °C for 4 min using an ultrasonic cavitation machine (MH-010S, Mxmoonant), and then the gel solutions stood at 35 °C for 1 min.

**Figure 7:**
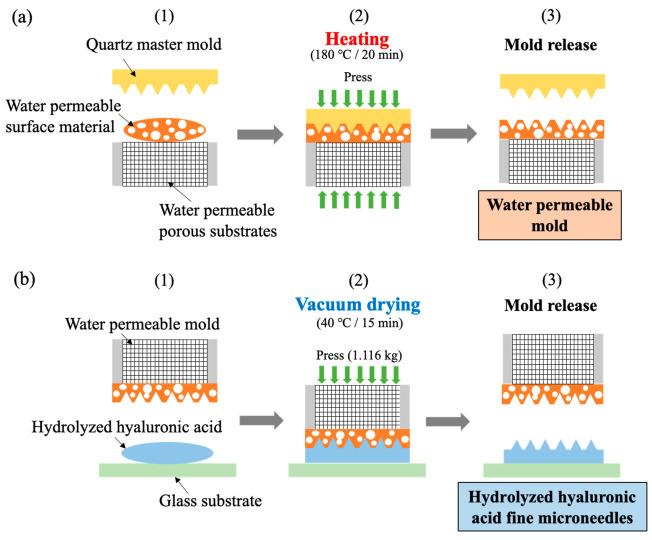
(**a**) First step of imprint lithography processes from quartz master mold with high-resolution microneedle pattern structures to the water permeable mold and (**b**) second step of vacuum environment imprint lithography processes from water permeable mold with inverted needle patterns to hydrolyzed hyaluronic acid gel solutions.

Figure 7a shows the first step of the imprint lithography processes from a quartz master mold (10 mm × 10 mm) with high-resolution microneedle pattern structures of 12 µm in diameter and 25 µm in height at a 78° angle to the water permeable mold. The process conditions were as follows. The quartz master mold used as the template was treated with a mold release agent (DURASURF DS-831TH, Harves, Japan) to release the quartz master mold from the water permeable surface material. (1) The water permeable surface material was placed on the water permeable porous substrates, which were ultrasonically cleaned with acetone for 10 min and then dried in the above vacuum dryer for 20 min. (2) The quartz master mold was placed in contact with the water permeable surface material, and imprinting was performed using a hand hot press digital (HHP-2D, AS ONE, Japan) under pressure at 180 °C for 20 min. (3) Once the heat was removed, the quartz master mold was released, and a water permeable mold with inverted needle patterns was obtained.

Figure 7b shows the second step of the vacuum environment imprint lithography processes, from the water permeable mold with inverted microneedle patterns to the hydrolyzed hyaluronic acid gel solutions. The process conditions were as follows. The glass substrates (S3233, MATSUNAMI, Japan) were hydrophilically treated for 5 min using UV ozone treatment equipment (LT0Z-180, Litho Tech Japan, Japan), to improve the adhesion between the hydrolyzed hyaluronic acid and the glass substrates. (1) Hydrolyzed hyaluronic acid gel solutions were placed on the hydrophilically treated glass substrate. (2) The water permeable mold was placed over the solutions, and pressure was applied with a metal weight (1.116 kg). Thereafter, it was dried in the vacuum dryer at 40 °C for 15 min to remove the water in the solutions through the water permeable mold. (3) The water permeable mold was then released to obtain the hydrolyzed hyaluronic acid fine microneedles. The hydrolyzed hyaluronic acid gel solutions were applied to 14 glass substrates under the condition of using one developed water permeable hybrid mold with inverted needle patterns. The pattern structures of the hydrolyzed hyaluronic acid fine microneedles and the referenced commercial microneedles were observed using a cold field emission SEM (Regulus8100, Hitachi High-Tech, Japan).

In addition, to confirm the dissolution behavior and basic properties of the hydrolyzed hyaluronic acid before and after the vacuum environment imprint lithography processes, three types of analysis of the hydrolyzed hyaluronic acid fine microneedles were carried out as follows.

1. Measurement of the dissolution behavior using environmental test equipment
 The evaluation of the dissolution behavior was performed for 35 min at 31 °C and 90–95% humidity using an incubator (PIC-101, AZ-ONE, Japan).
2. Measurement using a FT-IR
 The measurements were performed using an FT-IR (Spectrum Two; P erkin Elmer, USA). The measurement range was 450–4000 cm^−1^, the number of integrations was 4, and the resolution was 4 cm^−1^. The measurement was performed on films prepared by dropping hydrolyzed hyaluronic acid onto a glass slide and placing the slide at room temperature for 24 h. FT-IR measurements were carried out for component comparison before and after baking the films of hydrolyzed hyaluronic acid using a hot plate at 80 °C for 90 min.
3. Measurement using a Tg-DTA
 The measurement in nitrogen was performed using a Tg-DTA (EXSTAR TG/DTA7300, SII NanoTechnology, Japan) in the temperature range of 30 to 250 °C (temperature gradient: 10 °C /min). The weight of the analyzed sample was 5 mg.

## Data Availability

The datasets generated and/or analyzed during the current study are not publicly available because they belong to on-going research but are available from the corresponding author upon reasonable request.
